# A rapid VEGF-gene-sequence photoluminescence detector for osteoarthritis

**DOI:** 10.3389/fbioe.2024.1338901

**Published:** 2024-02-06

**Authors:** Hao Huang, Shuang Li, Xianjing Han, Yule Zhang, Lingfeng Gao, Xiangjiang Wang, Guiqing Wang, Zhi Chen

**Affiliations:** ^1^ Department of Orthopaedics, The Sixth Affiliated Hospital of Guangzhou Medical University, Qingyuan People’s Hospital, Guangzhou, China; ^2^ Key Laboratory of Optoelectronic Devices and Systems of Ministry of Education and Guangdong Province, Collage of Physics and Optoelectronics Engineering, Shenzhen University, Shenzhen, China; ^3^ College of Material Chemistry and Chemical Engineering, Key Laboratory of Organosilicon Chemistry and Material Technology, Ministry of Education, Hangzhou Normal University, Hangzhou, Zhejiang, China

**Keywords:** osteoarthritis, VEGF, photoluminescence, CRISPR/Cas12a, Mn-ZIF

## Abstract

Osteoarthritis (OA) has become a serious problem to the human society for years due to its high economic burden, disability, pain, and severe impact on the patient’s lifestyle. The importance of current clinical imaging modalities in the assessment of the onset and progression of OA is well recognized by clinicians, but these modalities can only detect OA in the II stage with significant structural deterioration and clinical symptoms. Blood vessel formation induced by vascular endothelial growth factor (VEGF) occurs in the early stage and throughout the entire course of OA, enables VEGF relating gene sequence to act as a biomarker in the field of early diagnosis and monitoring of the disease. Here in, a facile rapid detection of VEGF relating ssDNA sequence was developed, in which manganese-based zeolitic imidazolate framework nanoparticles (Mn-ZIF-NPs) were synthesized by a simple coprecipitation strategy, followed by the introduction and surficial absorption of probe ssDNAs and the CRISPR/Cas12a system components. Furthermore, fluorescence experiments demonstrated that the biosensor displayed a low detection limit of 2.49 nM, a good linear response to the target ssDNA ranging from 10 nM to 500 nM, and the ability of distinguishing single nucleotide polymorphism. This finding opens a new window for the feasible and rapid detection of ssDNA molecules for the early diagnose of OA.

## 1 Introduction

As the sixth leading cause of disability in the world, OA affects nearly 240 million people worldwide. Approximately 20% of the world’s population will be over 60 years old by 2050 according to the report of World Health Organization (WHO), in which 15% will have symptomatic OA, and one-third of these people will be severely disabled (Nelson, 2018). As a kind of chronic, debilitating joint disease, OA goes beyond anatomical and physiological degenerative alterations caused by cellular stress and degradation of the extracellular cartilage matrix begin with micro-and macro-injuries, which can be mainly characterized by joint degeneration with gradual loss of joint cartilage, bone hypertrophy, changes in the synovial membrane, and mechanical dysfunction (Gardner, 1983; Cicuttini and Wluka, 2014; Tiulpin et al., 2018; Abedin et al., 2019; Chen et al., 2019). Since OA is a very common disease, the early diagnose of it is of crucial importance for the purpose of providing affected patients with proper preventive treatments to delay the progression of OA disease, improve the long-term life quality of these patients, and alleviate the medical burden of human society (Chu et al., 2010; Chu et al., 2012; Iolascon et al., 2017). However, current clinical imaging techniques such as computed tomography (CT) and magnetic resonance imaging (MRI) can only detect OA in the II stage with significant structural deterioration and clinical symptoms (Möller et al., 2008; Braun and Gold, 2012; Roemer et al., 2014), while some biomolecular changes have already taken place prior to this irreversible stage (Debabrata and Sandell Linda, 2011). Articular cartilage (AC) is a layer of transparent cartilage primarily composed of cartilage matrix and chondrocytes located within the matrix lacunae, without any existence of blood vessels or lymphatic vessels. Oxygen molecules and nutrients needed by chondrocytes are supplied by surrounding synovial fluid and the underlying subchondral bone through the diffusion effect. Throughout one’s lifetime, AC maintains a hypoxic microenvironment, which can promote the proliferation and differentiation of chondrocytes and then benefit the synthesis of extracellular matrix. However, blood vessel formation is commonly observed in the AC of OA patients with higher expression level of VEGF in synovial fluid and blood. VEGF can activate endothelial cells and promote their proliferation and differentiation, thereby facilitating the generation of blood vessels in the synovium. Consequently, the formed vascular plexus can cover the surface of AC, preventing the chondrocytes from receiving nutrients from the synovial fluid, then lead to the degradation and destruction of AC (Jansena et al., 2012; Quan et al., 2014; Hamilton et al., 2016; Nagao et al., 2017). As mentioned above, the key role of VEGF playing in the development and progression of OA enables its relating gene sequence to act as a diagnostic marker of the disease.

**SCHEME 1:**
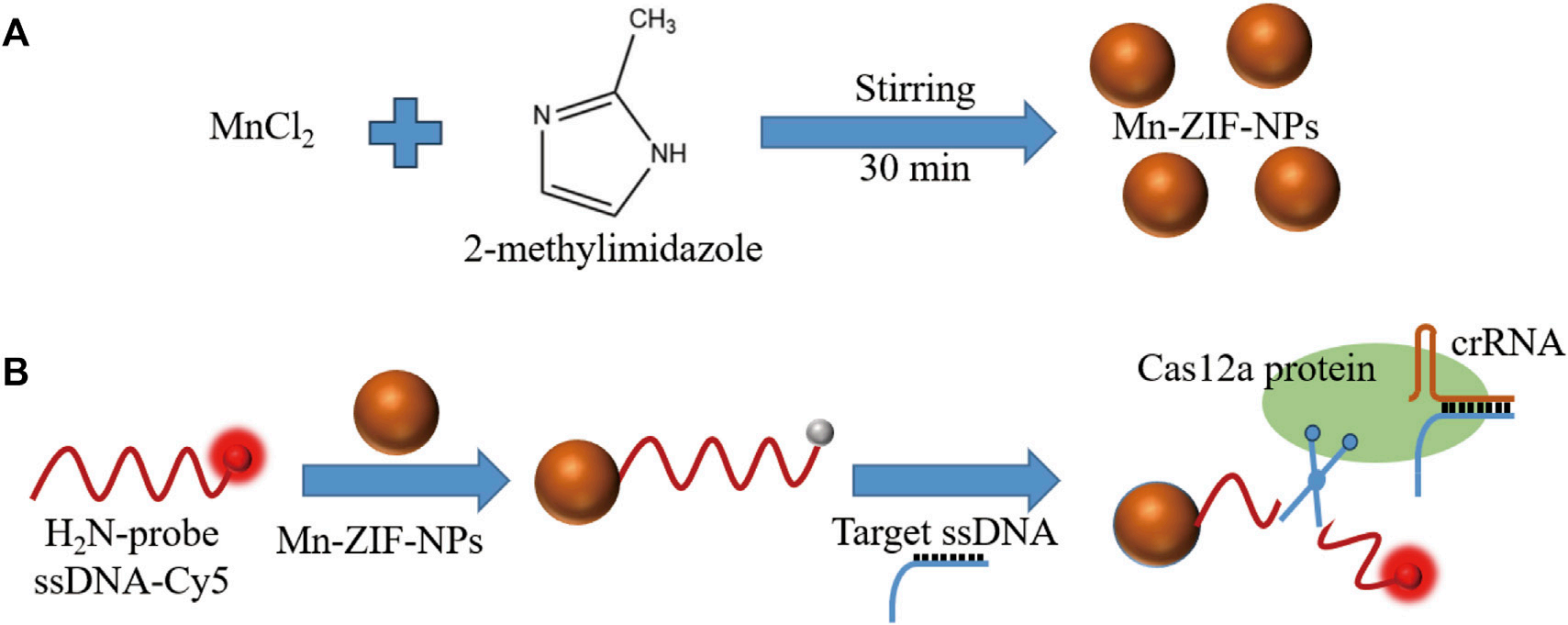
**(A)**. Fabrication and **(B)**. schematic diagram of Mn-ZIF-NPs-based CRISPR/Cas12a fluorescent-on type VEGF-related ssDNA sensing nanoplatform.

Clustered regularly interspaced short palindromic repeats (CRISPR)-Cas12a systems can recognize and indiscriminately cleave single-stranded DNA (ssDNA) upon recognition of the complementary DNA by a unique trans-cleavage effect (Chen et al., 2018; Pickar-Oliver and Gersbach, 2019). With the help of this unique trans-cleavage effect of Cas12a protein, CRISPR/Cas12a systems can bring better specificity and efficiency to the field of rapid and simple detection based on fluorescence analysis, which inspired our research (Li et al., 2018; Zhou et al., 2018; Xue et al., 2020; Chen et al., 2022; Wu et al., 2022). Herein, CRISPR/Cas12a and Mn-ZIF-NPs were synergistically driven to build a novel fluorescence-on type biosensor to achieve rapid detection of the VEGF gene sequences. Firstly, Mn-ZIF-NPs was prepared by a facile coprecipitation strategy (Scheme 1a). After that, the cyanine5 (Cy5)-labelled probe 5′-amino ssDNAs were added and adsorbed to the surface of as-prepared Mn-ZIF-NPs via the electrostatic interaction between the positive amino groups of probe ssDNAs and the negative surface of Mn-ZIF-NPs. The close proximity of probe DNA-Cy5 and Mn-ZIF-NPs led to fluorescence quenching of the dye, as the result of the fluorescence resonance energy transfer (FRET) from Cy5 to Mn-ZIF-NPs. After the addition of target VEGF-related DNA, the crRNA formed a duplex with this complementary ssDNA strand by hybridization, and then bound with the Cas12a protein to form a Cas12a-crRNA/ssDNA complex, which leads to the cleavage of probe DNA and the visual fluorescence recovering (Scheme 1b).

## 2 Materials and methods

### 2.1 Materials and instruments

The polyvinylpyrrolidone (PVP_24000_) was purchased from Aladdin Reagents. The manganese chloride tetrahydrate (MnCl_2_·4H_2_O, 99.99%) and 2-methylimidazole (98%) were both purchased from Macklin Inc. Oligonucleotides were purchased from Tsingke Biotech. Co. Ltd. (Beijing, China) with the sequence information shown in Table 1. Ultrapure water (18.25 MΩ⋅cm, 25°C) was used to prepare all solutions. All other chemicals used in this study were of analytical reagent grade and were used without further purification. The CRISPR/Cas12a assay kit purchasing from EZassay Biotech. Co. Ltd. (Shenzhen, China) was used in accordance with the manufacturer’s guidelines unless otherwise stated. The ultrasonication cleaner, Elmasonic S 60, was purchased from Elma Co. Ltd., Germany. The centrifuge used in this work was a Sigma 3-30k (Sigma, Germany). Fluorescence photos were taken by Quantum ST5 (Vilber Lourmat Co. Ltd., France) and the corresponding quantitative measurements were performed with an LightCycler 480II (Roche Ltd., Switzerland). HRTEM images and EDS Mapping data were both taken under a Talos F200X microscope (FEI Electronics, U.S.A.). AFM images were obtained with Demension icon and XRD data were collected using a D8 Advance instrument (both Bruker, Germany). UV-VIS-NIR curve was measured by UH4150 (Hitachi, Japan). Zeta potential was taken by an Zetasizer Nano ZS90 (Malvern, England).

**TABLE 1:** Sequence information of oligonucleotides used in the experiments, and mutated sites are presented as bold letters and labelled as red.

Name	Sequence (5′-3′)
probe ssDNA	Cy5—TTATT—NH_2_
crRNA	UAA UUU CUA CUA AGU GUA GAU UCU GAG UCG GAG GCU GUG GU
target ssDNA	AGG GCA GGG CCC ACC ACA GCC TCC GAC TCA GAG GAA GAG GCT GCC CTG CAA GGA GGC CTC
M11	AGG GCA GGG CCC ACC ACA GCC **A**CC GAC TCA GAG GAA GAG GCT GCC CTG CAA GGA GGC CTC
M12	AGG GCA GGG CCC ACC ACA GCC TCC G**C**C TCA GAG GAA GAG GCT GCC CTG CAA GGA GGC CTC
M13	AGG GCA GGG CCC ACC ACA GCC TCC GAC **C**CA GAG GAA GAG GCT GCC CTG CAA GGA GGC CTC
M2	AGG GCA GGG CCC ACC ACA GCC TCC GAC TC**T** **C**AG GAA GAG GCT GCC CTG CAA GGA GGC CTC
M3	AGG GCA GGG CCC ACC ACA GCC **A**CC GAC TC**T** **C**AG GAA GAG GCT GCC CTG CAA GGA GGC CTC
NC	AAA TGG CGA ATC CAA TTC CAA GAG GGA CCG TGC TGG GTC ACC CGC CCG GGA ATG CTT CCG

Mutated sites are presented as bold letters and labelled as red.

### 2.2 Material preparation

In brief, 500 mg PVP_24000_ was dissolved in 20 mL ultrapure water with continuous stirring of 500 rpm, followed by the addition of MnCl_2_·4H_2_O aqueous solution (1 M, 500 μL) and stirring for 3 min. Meanwhile, 2-methylimidazole aqueous solution (1 M, 1 mL) was diluted into 20 mL ultrapure water and then this diluted solution was injected into the abovementioned MnCl_2_·4H_2_O aqueous solution, followed by another stirring for 30 min. The resulting suspension was collected and centrifuged at 25000 × g for 2 min, and the precipitation was been washed with ultrapure water for several times. Finally, the as-prepared product was re-dispersed into ultrapure water, then was sealed and stored in a dark environment at room temperature.

### 2.3 Fluorescent assay of DNA detection

The fluorescent measurements were carried out in 8-strip PCR tubes at 37°C. At the first, 10× reaction buffer (2 μL), Cas12a protein (2 μM, 1 μL) and crRNA (4 μM, 1 μL) were mixed in RNase-free water and incubated at 37°C for 15 min, followed by the addition of various concentration of VEGF-related target ssDNA or mismatch ssDNA (1 μL) and then another incubation at 37°C for 15 min. After that, various concentration of Mn-ZIF-NPs and probe Cy5-ssDNA-NH_2_ (10 μM, 1 μL) were added with the final volume of 20 μL filled with RNase-free water. Finally, the fluorescence measurement was performed at 37°C.

## 3 Results and discussions

### 3.1 Material characterization

**FIGURE 1:**
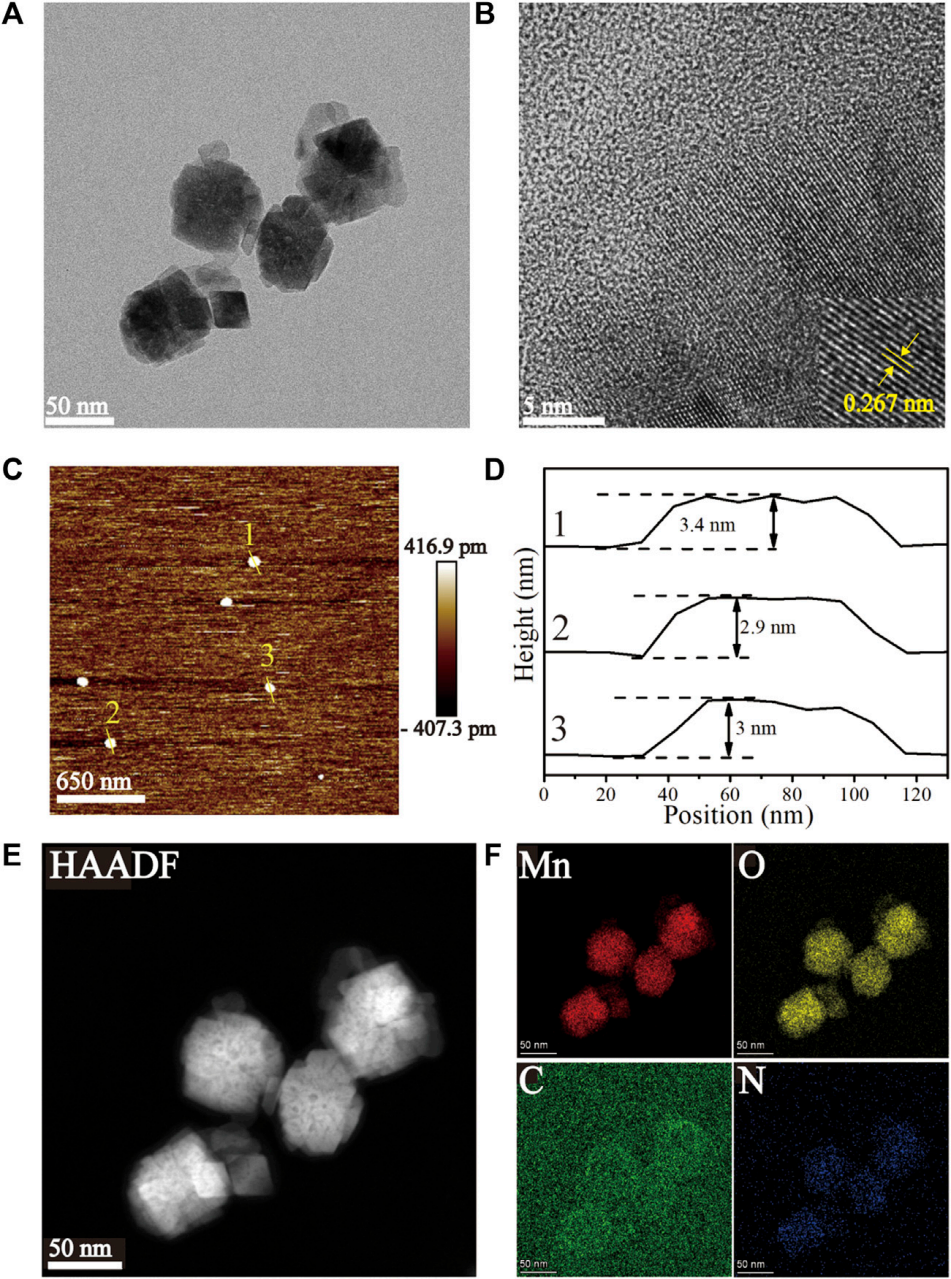
Characterization of the as-prepared Mn-ZIF-NPs. **(A, B)** HRTEM images, **(C, D)** AFM images, **(E, F)** EDS Mapping images.

The HRTEM images (Figures 1A, B) of the Mn-ZIF-NPs reveal irregular nanocrystals with a mean diameter of about 50 nm and clear lattice fringes of 0.267 nm. The AFM images (Figures 1C, D) indicate that the average thickness of NPs was less than 3.5 nm. EDS element mapping in Figures 1E, F confirms the uniformly distribution of Mn(red), O(yellow), C(green) and N(blue) elements inside the NPs.

**FIGURE 2:**
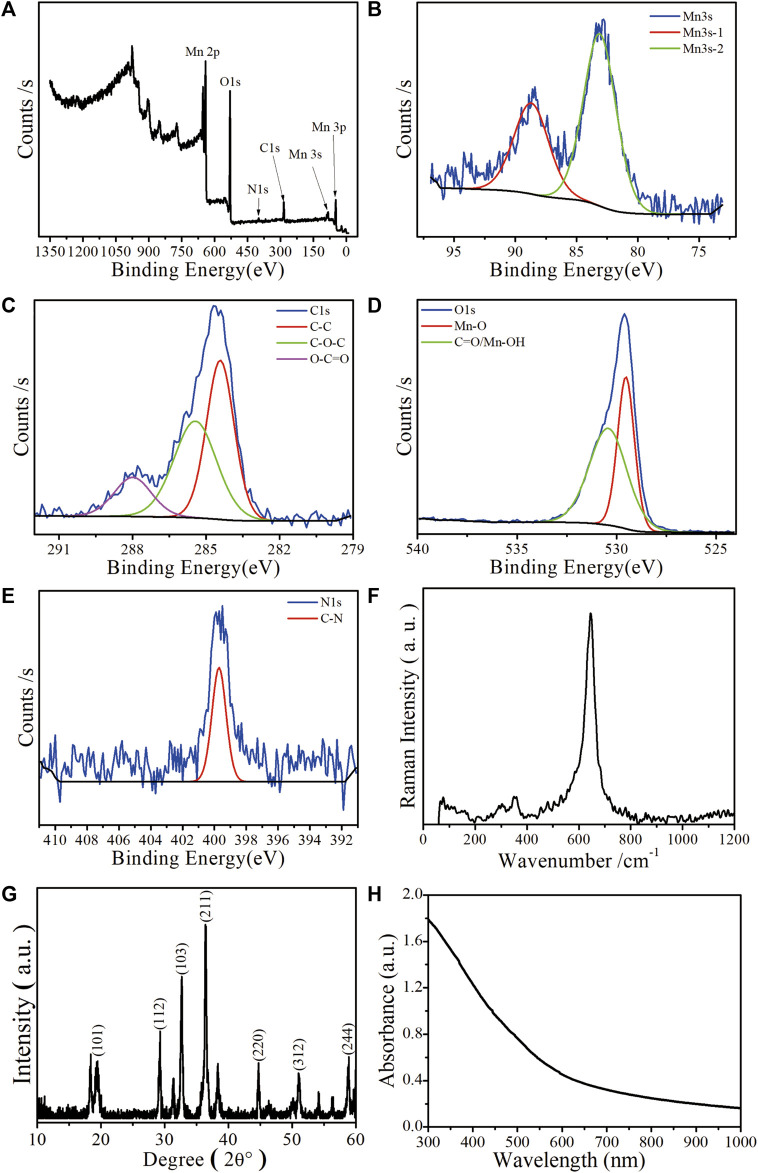
Characterization of the as-prepared Mn-ZIF-NPs. **(A)** XPS spectrum, **(B)** Mn 3s, **(C)** C1s, **(D)** O1s, **(E)** N1s. **(F)** Raman spectrum, **(G)** XRD pattern and **(H)** UV-VIS-NIR curve.

Mn-ZIF-NPs were subjected to XPS analysis to find the elemental states and the result is shown in Figures 2A–D. The observed Mn 3s binding energy values are 83.2 eV and 88.7 eV with two multiple split components caused by coupling of non-ionized 3s electron with 3d valence-band electrons. The ΔE of 5.5 eV is in the range between 6.0 eV (MnO, Mn^2+^) and 5.3 eV (Mn_2_O_3_, Mn^3+^), indicating that the Mn was observed in multiple oxidation states in the imidazolate framework (Biesinger et al., 2011). Besides, the carbon functionalities like C-C, C-O-C, and O-C=O were observed at 283.4 eV, 285.4 eV and 288 eV, respectively (Shchukarev and Korolkov, 2004). Furthermore, the O 1s spectrum of 529.5 eV and 530.4 eV correspond to Mn−O and C=O/Mn-OH, respectively (Figure 2D). The N 1s XPS spectrum in Figure 2E reveals the existence of 399.7 eV corresponding to C-N. In Figure 2F, the major Raman peak in the spectra of Mn-ZIF-NPs at 642.8 cm^−1^ can be ascribed to the Mn^2+^/^3+^ coordinated with the imidazolate framework. As is shown in Figure 2G, the X-ray diffraction (XRD) peaks of Mn-ZIF-NPs well indexed to coincide with other work (Sankar Selvasundarasekar et al., 2022). The relative high absorption intensity in the VIS region enables the Mn-ZIF-NPs to efficiently quench the fluorescence of our probe ssDNA (Figure 2H), while the zeta potential peak of Mn-ZIF-NPs at −3.26 mV makes the surface absorption of probe DNA possible.

### 3.2 Optimization of experimental conditions

**FIGURE 3:**
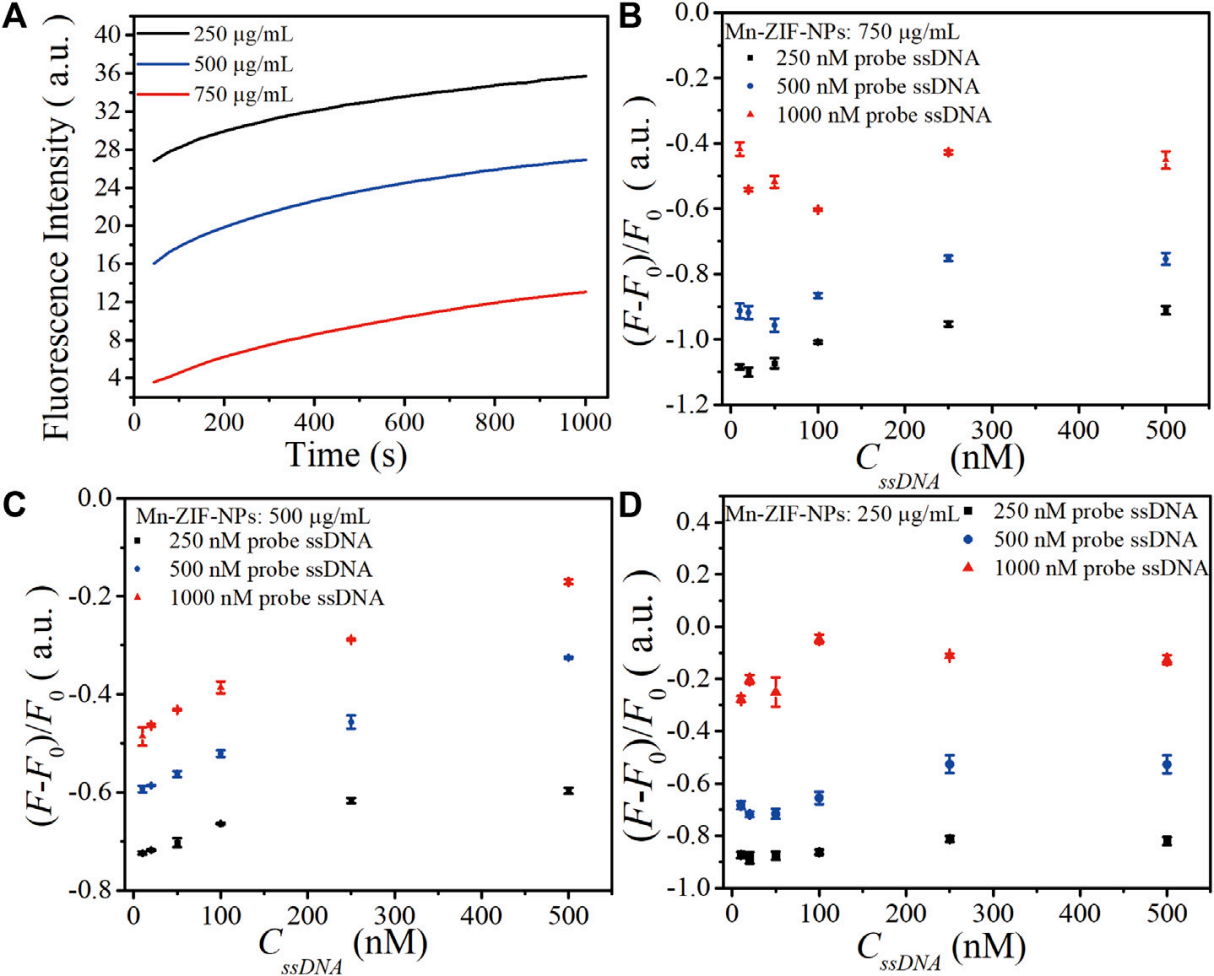
**(A)**. The fluorescence curve of probe ssDNA in the presence of Mn-ZIF-NPs at different concentrations (750, 500 and 250 μg/mL) after incubation with target VEGF-related ssDNA (100 nM) for 15 min at 37°C. **(B)** The calibration relationship of *ΔF* versus the concentration of target ssDNA (*C*_*ssDNA*_) when the Mn-ZIF-NPs concentration was 750 μg/mL. **(C)** The calibration relationship of *ΔF* versus *C*_*ssDNA*_ when the Mn-ZIF-NPs concentration was 500 μg/mL. **(D)** The calibration relationship of *ΔF* versus *C*_*ssDNA*_ when the Mn-ZIF-NPs concentration was 250 μg/mL. The curves represent the average values of three wells with error bars showing the standard deviation of three wells at each condition.

The concentration of probe ssDNA and Mn-ZIF-NPs were optimized to make the performance of the biosensor best. The tested probe ssDNA concentration varied from 250 to 1,000 nM, and three concentrations of Mn-ZIF-NPs (750, 500 and 250 μg/mL) were compared after incubation with target VEGF-related ssDNA (100 nM) for 15 min at 37°C. Figure 3A shows the fluorescence curve of probe ssDNA in the presence of Mn-ZIF-NPs at different concentrations, illustrating that the quenching efficiency of probe ssDNA become higher as the concentration of Mn-ZIF-NPs increased, possibly due to the increased amount of probe ssDNA absorbed on Mn-ZIF-NPs. The results for target ssDNA detection were presented in Figures 3B–D. When the concentration of Mn-ZIF-NPs was 750 μg/mL, no linear interval for target ssDNA detection can be observed. Figure 3C shows that there is a linear relationship between *ΔF* (*ΔF* = *F*/*F*_*0*_—1, *F*_*0*_ is the fluorescence intensity of the probe Cy5-ssDNA, *F* is the fluorescence intensity of the probe Cy5-ssDNA/Mn-ZIF-NP complex after incubating with different concentrations of target ssDNA) and *C*_*ssDNA*_ in the concentration range of 10 to 500 nM for 500 nM probe ssDNA, 20 to 100 nM for 250 nM probe ssDNA and 10 to 100 nM for 1,000 nM probe ssDNA when the concentration of Mn-ZIF-NPs was 500 μg/mL, respectively. However, when the concentration of Mn-ZIF-NPs further reduces to 250 μg/mL, no linear interval for target ssDNA detection can be observed again (Figure 3D). With the aim of getting clear linear relationship with widest detection range, 500 nM of probe ssDNA and 500 μg/mL of Mn-ZIF-NPs were employed for target ssDNA detection.

### 3.3 DNA sensing performance of Mn-ZIF-NPs

**FIGURE 4:**
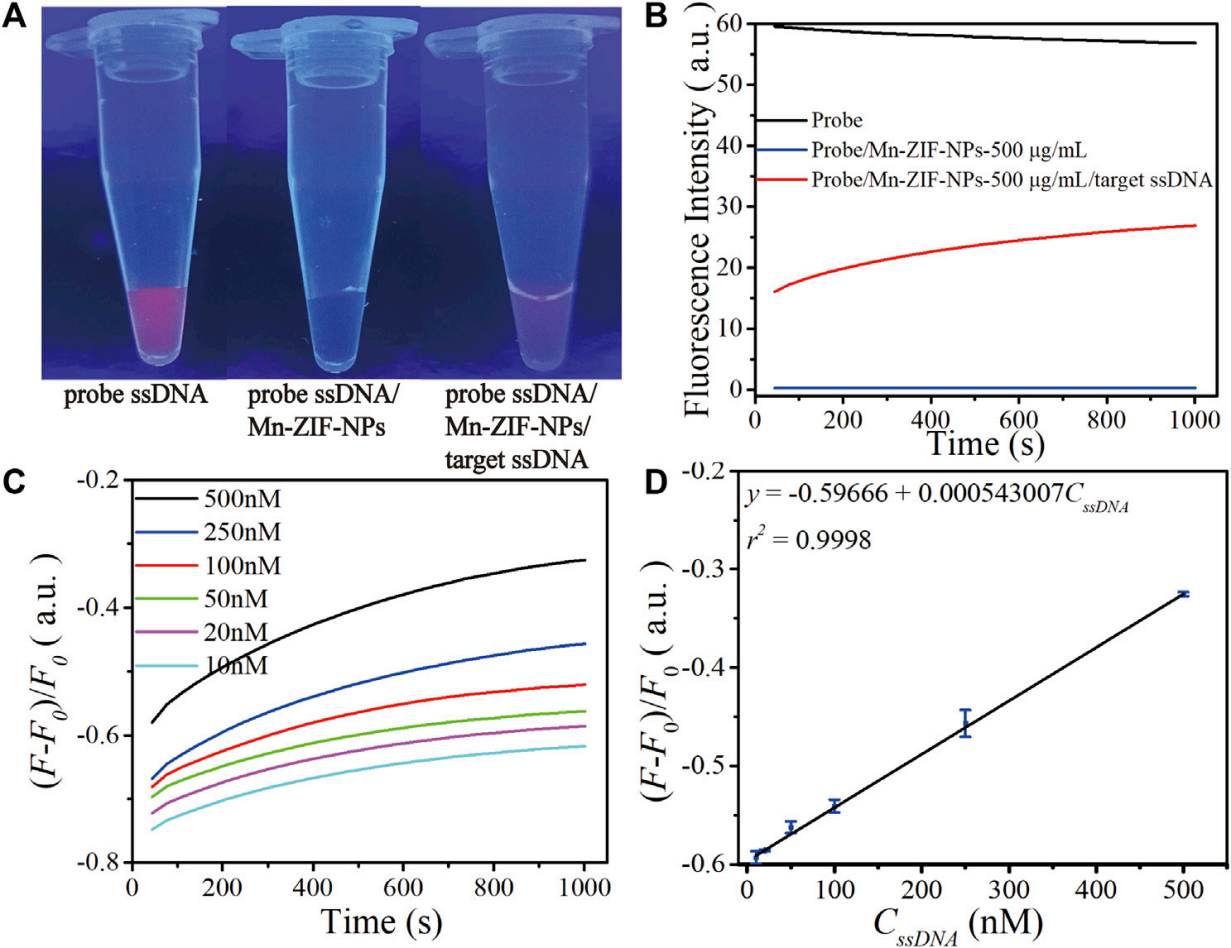
Performance of as-prepared ssDNA sensor. **(A)** Fluorescence image of the CRISPR/Cas12a system including probe Cy5-ssDNA solution (500 nM) before and after mixing with Mn-ZIF-NPs (500 μg⋅mL^−1^), the Cy5-ssDNA/Mn-ZIF-NP complex after incubation with target VEGF-related ssDNA (100 nM) for 15 min at 37°C. **(B)** Corresponding fluorescence spectra of the probe Cy5-ssDNA (black curve), Cy5-ssDNA/Mn-ZIF-NPs (blue curve) and Cy5-ssDNA/target ssDNA/Mn-ZIF-NPs (red curve). **(C)** The fluorescence emission spectra of the probe ssDNA after incubating with varying concentrations of target ssDNA at 10, 20, 50, 100, 250 and 500 nM for a total of 30 min. **(D)** The calibration curve of OD versus *C*_*ssDNA*_ (10–500 nM). The curves represent the average values of three wells with error bars showing the standard deviation of three wells at each concentration.

Figure 4A depicts fluorescence photos of the probe Cy5-ssDNA solution, as the strong fluorescence dramatically quenched after the addition of Mn-ZIF-NPs with the final concentration of 500 μg⋅mL^−1^, confirming the efficient quenching capability of the Mn-ZIF-NPs. As shown in Figure 4C, the addition of the target VEGF related ssDNA followed by a total of 30 min’ incubation at 37°C leads to obvious fluorescence recovery even can be directly observed by naked eyes, which is primarily due to the cleavage of probe ssDNA by Cas12a-crRNA/target ssDNA complex and the releasing of Cy5 from the surface of Mn-ZIF-NPs.

To validate the abovementioned naked-eyes observation more precisely, we performed the fluorescence spectroscopy measurement shown in Figure 4B. The initial value of strong emission of probe Cy5-ssDNA at 670 nm is 56.8 a.u. (black curve). After the addition of Mn-ZIF-NPs, the fluorescence intensity is rapidly quenched to 0.29 a.u. (blue curve). Due to the cleavage of probe ssDNA by Cas12a-crRNA/target ssDNA complex, the fluorescence intensity is greatly increased to 26.9 a.u. after the addition of target ssDNA (red curve).

To examine the sensitivity of this nanoplatform, different concentrations of ssDNA were introduced and then a progressive increase in the fluorescence intensity at λ = 670 nm with increasing ssDNA concentration is observed (Figure 4C), which exhibits a linear response in the concentration range of 10 nM–500 nM. The linear regression equation is *ΔF* = −0.59666 + 0.000543007⋅*C*_*ssDNA*_ (nM, *r* ^*2*^ = 0.9998). The detection limit is 2.49 nM as estimated according to the 3σ rule (Figure 4D).

**FIGURE 5:**
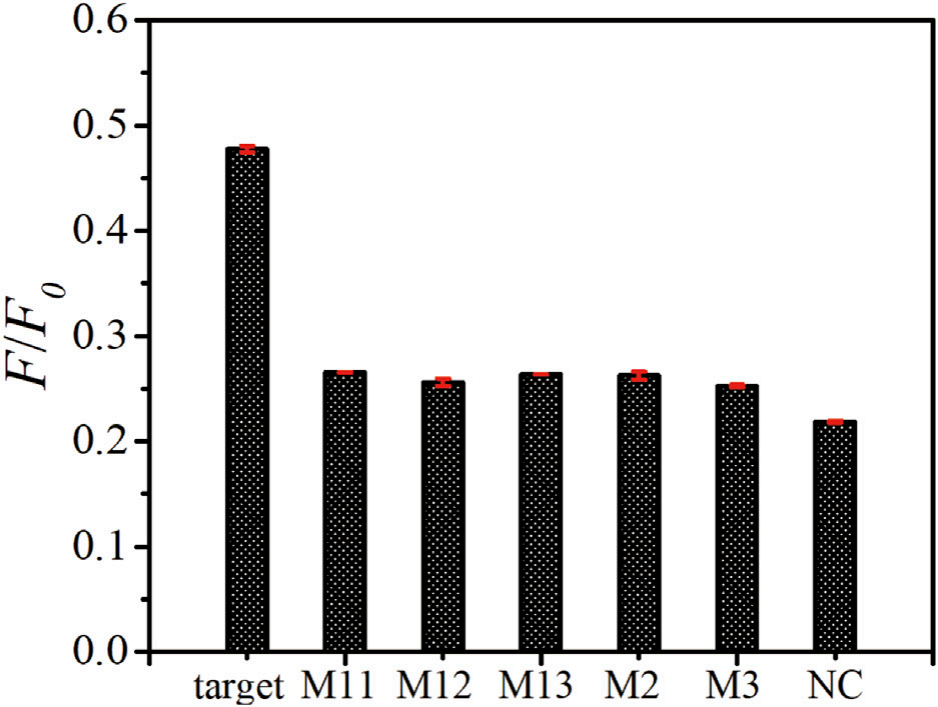
Specificity assay of as-prepared ssDNA sensor. The fluorescence emission spectra upon the addition of target ssDNA, M11, M12, M13, M2, M3, and NC with the same concentration of 100 nM. The error bars represent the standard deviation of three wells under the same conditions.

To study the selectivity of this platform, a comparison of the fluorescence recovery responses of the target ssDNA, mismatched ssDNA (M11, M12, M13, M2, M3) and non-complementary ssDNA (NC) was performed. As shown in Figure 5, the *F*/*F*_*0*_ value obtained upon the incubation of 100 nM target ssDNA, M11, M12, M13, M2, M3 and NC are 0.477, 0.266, 0.256, 0.264, 0.262, 0.252 and 0.218, respectively. The fluorescence intensity ratios of all the mismatched group don’t show obvious differences, which demonstrated that our biosensor could distinguish the target ssDNA from single-base mismatched or non-complementary ones.

### 3.4 Additional specificity test

We also tested the responses of biosensor after mixed target ssDNA with M11, M2, M3 and NC, respectively. In our experiments, the detected concentrations of “100 nM target”, “100 nM target mixed with 40 nM M11”, “100 nM target mixed with 40 nM M2”, “100 nM target mixed with 40 nM M3” and “100 nM target mixed with 40 nM NC” are calculated by the linear equation of biosensor. As can be seen from Table 2, the accuracies are in the range of 90% to 110%, which demonstrated that the signal of target ssDNA remains almost the same in the presence of other ssDNAs. The results further verify the specificity of the biosensor.

**TABLE 2:** The responses of biosensor to mixed samples.

Samples	Detected concentration (nM)	Accuracy (%)
100 nM target	108.67	108.67
100 nM target + 40 nM M11	95.93	95.93
100 nM target + 40 nM M2	94.17	94.17
100 nM target + 40 nM M3	94.03	94.03
100 nM target + 40 nM NC	97.92	97.92

## 4 Conclusion

In summary, we built a simple and fast CRISPR/Cas12a-based VEGF related ssDNA detection nanoplatform by employing Mn-ZIF-NPs prepared through facile nanoprecipitation strategy as fluorescence quenching material. The biosensor had a linear range of 10–500 nM, a detection limit of 2.49 nM, with excellent specificity of single nucleotide polymorphism. It is therefore envisaged that our research paves the way for further investigations and applications of CRISPR/Cas12a system and nanomaterials in fluorescence quenching-based biosensing for biomedical research and particular the early clinical diagnostics of OA.

## Data Availability

The datasets presented in this study can be found in online repositories. The names of the repository/repositories and accession number(s) can be found in the article.
