# Corrigendum: A rapid VEGF-gene-sequence photoluminescence detector for osteoarthritis

**DOI:** 10.3389/fbioe.2024.1385924

**Published:** 2024-03-14

**Authors:** Hao Huang, Shuang Li, Xianjing Han, Yule Zhang, Lingfeng Gao, Xiangjiang Wang, Guiqing Wang, Zhi Chen

**Affiliations:** ^1^ Department of Orthopaedics, The Sixth Affiliated Hospital of Guangzhou Medical University, Qingyuan People’s Hospital, Guangzhou, China; ^2^ Key Laboratory of Optoelectronic Devices and Systems of Ministry of Education and Guangdong Province, Collage of Physics and Optoelectronics Engineering Shenzhen University, Shenzhen, China; ^3^ College of Material Chemistry and Chemical Engineering, Key Laboratory of Organosilicon Chemistry and Material Technology, Ministry of Education, Hangzhou Normal University, Hangzhou, Zhejiang, China

**Keywords:** Osteoarthritis, VEGF, photoluminescence, CRISPR/Cas12a, Mn-ZIF

In the published article, there were errors in section **3 Results and discussions**, *3.3 DNA sensing performance of Mn-ZIF-NPs*, Paragraph two.

In the original article, the sentence reads:

“The initial value of strong emission of probe Cy5-ssDNA at 670 nm is 69 a.u.”

This should have been written as:

“The initial value of strong emission of probe Cy5-ssDNA at 670 nm is 56.8 a.u.”

In the original article, the sentence reads:

“Due to the cleavage of probe ssDNA by Cas12a-crRNA/target ssDNA complex, the fluorescence intensity is greatly increased to 33 a.u. after the addition of target ssDNA.”

This should have been written as:

“Due to the cleavage of probe ssDNA by Cas12a-crRNA/target ssDNA complex, the fluorescence intensity is greatly increased to 26.9 a.u. after the addition of target ssDNA.”

The authors apologize for these errors and state that this does not change the scientific conclusions of the article in any way. The original article has been updated.

